# GASTRIC SCHWANNOMA: THE GIST SIMULATOR

**DOI:** 10.1590/0102-672020210003e1590

**Published:** 2021-12-17

**Authors:** Manuel FIGUEROA-GIRALT, Omar ORELLANA, José Manuel HERRANZ

**Affiliations:** 1 Department of Surgery, Clinical Hospital of the University of Chile, Santiago, Chile; 2 Department of Pathology, Clinical Hospital of the University of Chile, Santiago, Chile

**Keywords:** Gastric tumor, Gastric schwannoma, Neurilemoma, Mesenchymal tumor, Laparoscopic surgery, Tumor gástrico, Schwannoma gástrico, Neurilenoma, Tumor mesenquimal, Cirurgia laparoscópica

## INTRODUCTION

Schwannomas also known as neurilemoma, are rare tumors that emerge from peripheral nervous system, particularly Schwann cells. The gastrointestinal location is very rare being the stomach the most affected organ. However, gastric schwannoma represents only 0,2% of all gastric tumors. Usually they have benign prognosis, but malignant transformation has been described. Symptoms are usually vauge and depend on the localization^1^ ^,^ ^3^ ^,^ ^4^ ^,^ ^11^. 

In this case report we present a patient with and antral submucosal gastric tumor that recived totally laparoscopic resection. 

## CASE REPORT

A 56-year-old woman, with hypertension, hypothyroidism, clinical history of six months of epigastric pain, without vomits, dysphagia, jaundice, fever or weight loss, and no specific founding at physical examination, presented for consultation. 

**FIGURE 1:**
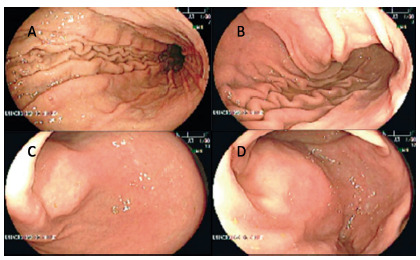
Upper gastrointestinal endoscopy: A) gastric body; B) impression of submucosal tumor at de anterior surface of gastric angle; C and D) antral submucosal tumor without mucosal erosion.

The upper endoscopy shown an anterior antral submucosal tumor of 5 cm, with no ulceration (Figure 1). 

**FIGURE 2:**
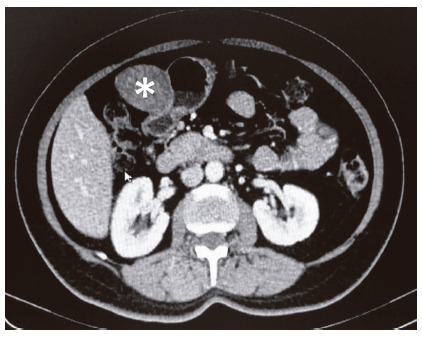
Pre-operative axial image of abdominopelvic CT: *) anterior antral solid exophytic tumor of 5.2x4.8 cm

The abdominopelvic CT scan showed an anterior antral submucosal exophytic solid tumor of 5.2x4.8 cm, without celomic, lymphatic or visceral metastasis (Figure 2).

**FIGURE 3:**
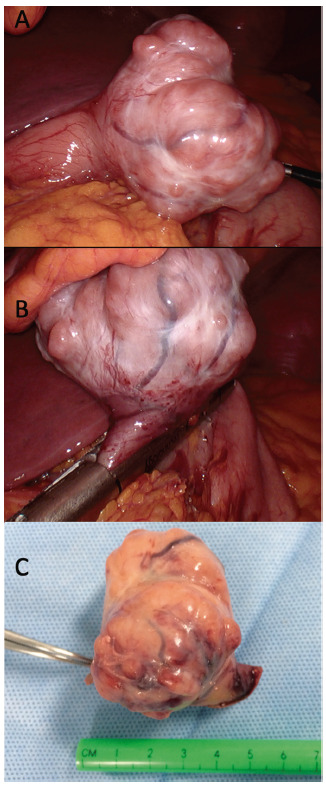
Surgical aspects: A) anterior antral exophytic tumor with expansive growing; B) resection of the tumor with mechanical suture with laparoscopic stapler; C) tumor resected.

With the diagnosis of an antral GIST, the patient underwent laparoscopic exploration and resection of the lesion. A 15-mmHg pneumoperitoneum and four trocars (one of 5 mm and three of 12 mm) were used. A 6 cm exophytic gastric tumor was found in the same position describe by the endoscopy and CT. Dissection of the grater curvature was performed to access the lesser omentum and posterior wall of the stomach. After passing a 36F bogie throw the pylorus, a total resection of the tumor was performed with two staplers (Figure 3 and video1).

**VIDEO 1:**
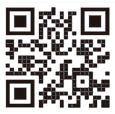
GASTRIC SCHWANNOMA: THE GIST SIMULATOR

https://www.youtube.com/watch?v=2epiw-x9Mig

The patient had a good recovery from surgery, initiating oral intake at 24 h after surgery and was dismissed at the 3^th^ postoperative day with no postoperative complications. 

**FIGURE 4:**
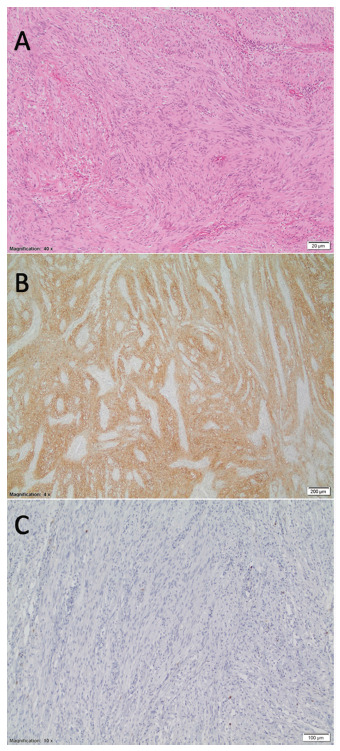
Histologyc examination: A) mesenchymal neoplasia composed of fused cells with elongated nuclei and sharp edges with little pleomorphism and mitotic count from 0 to 2/50 HPFs and cells in areas are arranged forming palisades (H&E 40x); B) intense and diffuse positive in cells under study (S100); C) negative in cells under study (CD117).

The histopathologic examination showed a 6.2x5 cm gastric schwannoma, with 0-2/50 field mitotic count, non-necrosis, free margin, S100 highly positive, actine negative, desmin negative, CD34 negative, CD 117 negative, COG1 negative, KI67 nuclear positive in 5% of cells (Figure 4).

**FIGURE 5:**
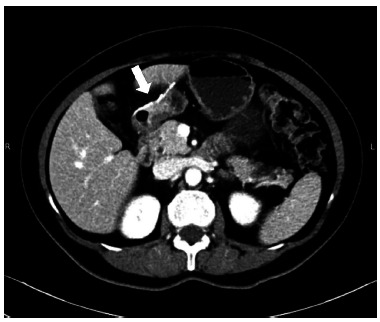
Postoperative axial image of abdominopelvic CT (arrow=tumor resected)

After discussion at the oncology committee, a surveillance conduct was decided. At eight months follow up the patient had no signs of surgical or oncologic complication (Figure 5).

## DISCUSSION

Submucosal gastrointestinal tumors have three different histological groups: GIST, myogenic (leiomyomas or leiomyosarcomas), and neurogenic tumors (schwannomas, granular cell tumors and neurofibromas). In the neurogenic group 91% are scwhannomas, and the most frequents locations are stomach (60-70%) and colorectal. Gastric schwannoma represents 5% of nonepithelial gastric tumors^8^ ^,^ ^9^ ^,^ ^11^.

As described by Bruneton^1^ and Mekras^8^ the majority of the patients with gastric schwannoma are women, between the 6-7^th^ decade of life. Usually they are asymptomatic, but when they require surgery, 42-88% have abdominal pain, 25-65% gastrointestinal bleeding and 10-25% weight loss. The localization is usually at the body (50%) or antrum (32%) and the size is less than 10 cm in 88% of cases, and less than 5 cm in 48% of them. In our case report, this demographic, clinical and anatomical characteristics are present. 

Endoscopic and tomographic study are the first line approach, but they cannot differentiate accurately between GIST, myogenic and neurogenic tumors^11^. A second line of study is the endoscopic ultrasound with guided biopsy, but the cost and availability should be consider, especially if the lesion is located at the gastric wall, which usually has in experience groups a comfortable surgical access and low morbimortality in case of resection. In our case the preoperative diagnosis was a presumed GIST, principally because is the most frequent gastric submucosal mesenchymal tumor. Considering the symptoms and little additional benefit of endoscopic ultrasound, we decided to perform a total resection of the tumor.

Surgical therapy is the cure in most cases without any other adjuvant therapy. The surgical approach depends on the tumor size, localization, surgeon experience and preference. The most important thing is to achieve negative margins, and to do so, resections could be from an economic enucleation and suture, to a partial or total gastrectomy. Because of the nature and frequency of this tumor, the literature available consist in case reports^1^ ^,^ ^8^, case series^3^ ^,^ ^14^, case-controls studies^4^ and reviews^6^ ^,^ ^9^ ^,^ ^11^ ^,^ ^14^. Many of the reports doesn’t declare the surgical approach, some of them specified a laparoscopic one^8^ ^,^ ^13^. As we mentioned before, in trained groups a laparoscopic approach with hemostatic sealant technology and staplers, are comfortable, short and safety procedures, that also contribute with all the benefit of minimally invasive surgery^2^. 

The pathologic examination is the key to differentiate between all subtypes of submucosal gastric tumors. Before the advances in immunohistochemical stain, the majority of this tumors were diagnosed as leiomyoma and leiomyosarcoma, but now with the advent of c-Kit, DOG1 and CD34 which are usually positive in GIST, SMA and desmin positive in leiomyoma, S100 positive in schwannoma.

After free margin surgery the prognosis is excellent, some argue that the risk of malignant transformation is theorical, and that histologic findings such as tumor size and mitotic rate have no prognostic significance^8^ ^,^ ^14^, but since malignant schwannoma have been described^1^ ^,^ ^7^ ^,^ ^10^ ^,^ ^12^ and the follow-up in tumors larger than 10 cm or with a mitotic rate >10/50 HPFs is limited, a clinical, endoscopic and tomographic surveillance should be consider according to each patient^14^. 
